# Gambling marketing in Finland: Experiences of exposure and regulation among Finnish individuals who gamble regularly

**DOI:** 10.1177/14034948251394913

**Published:** 2025-12-17

**Authors:** Paula P. Jääskeläinen, Eveliina V. Väkeväinen, Virve K. Marionneau

**Affiliations:** 1 University of Helsinki, Faculty of Social Sciences, Centre for Research on Addiction, Control and Governance, Finland

**Keywords:** Gambling, marketing, advertising, Finland, public health campaigns

## Abstract

**Aims:**

Gambling marketing can increase intentions to gamble, gambling consumption and gambling harms. In Finland, a substantial part of the population is at risk of gambling problems. Currently, the Finnish gambling marketing environment is changing: public health concerns have resulted in recent restrictions in gambling marketing as well as other measures to reduce gambling harm. However, the upcoming licensing system will soon significantly increase the availability and visibility of gambling by liberalising regulations. This study focuses on exposure to marketing and opinions on marketing regulations in a country with a changing regulatory landscape.

**Methods:**

This study presents results of an online survey (*N*=132) amongst Finnish individuals who gamble regularly, focusing on perceptions and experiences with gambling marketing. The quantitative data are analysed using cross-tabulation and bivariate correlations, and the qualitative data by using thematic content analysis.

**Results:**

Despite recent marketing restrictions, participants were widely exposed to and identified various forms of marketing by both the monopoly and offshore operators. Marketing by offshore providers was described as widespread and aggressive. The monopoly operator actively leveraged public health messaging as a form of marketing. Of the participants, 60.5% indicated wanting stricter marketing regulations or a blanket ban on gambling marketing.

**Conclusions:**

**Gambling marketing is ubiquitous in Finland. Those who gamble are widely exposed to different forms of marketing from both the monopoly and offshore providers. The upcoming licensing system is likely to further increase the visibility of marketing. Many consumers prefer stricter regulation owing to public health concerns.**

## Introduction

Gambling has become a pressing public health issue globally. Increasing availability and visibility of gambling, particularly in digital environments, causes harm to individuals, families and communities [1]. According to a recent meta-analysis, 8.7% of people worldwide gamble at any risk level, while 1.4% of individuals experience problem gambling. Any risk refers to scoring at least one point on screening instruments for gambling problems; problem gambling requires a higher score depending on the instrument used [2]. In Finland, the 2023 population study found that 14.2% of the population gambled at any risk level and 2.3% had a gambling problem [3]. Gambling causes a wide range of harms, including emotional distress, health and relationship problems, and financial difficulties [3,4].

Gambling-related harms are driven by commercial practices of the gambling industry [1]. From a consumer perspective, marketing is amongst the most visible commercial strategies. A growing body of research shows that marketing increases gambling intentions, engagement, and gambling-related problems [5–8]. For example, sports sponsorship plays a significant role in increasing exposure, purchase intentions and consumption of gambling products, with a particular appeal to children and young people [9]. Direct forms of marketing have been shown to induce unplanned spending among existing customers and those already experiencing gambling problems [10–12]. Gambling marketing can also be difficult to identify, especially on social media. Gambling operators post non-gambling-related content that followers, particularly children and adolescents, might not recognise as marketing [13]. Similarly, operators can leverage ‘responsible gambling’ messages as marketing strategies [14,15]. The current study focuses on experiences of gambling marketing in Finland. Finland provides a good case example for understanding contemporary gambling marketing practices and the impacts of changing regulations. Currently, the provision and marketing of gambling are legal only for the national gambling monopoly Veikkaus. Veikkaus marketing has for long been firmly embedded in everyday environments and emphasised the monopoly’s societal role [16–18]. In addition, numerous unlicensed offshore operators target marketing at Finnish consumers [19]. Although offshore gambling marketing in Finland is, in principle, illegal, it is difficult to prevent, while it also increases overall exposure to advertising and harm [20,21].

Finland has attempted to reduce marketing exposure via stricter regulation. In 2021, following increased public health concerns [22], new marketing provisions and mandatory identification were introduced into Finnish gambling legislation. Since these changes, Veikkaus has adjusted its commercial communications and reduced its marketing budget from €47.3m in 2018 to €24.6m in 2023 [23,24]. In 2022, the bulk of Veikkaus visibility consisted of campaigns encouraging individuals to adopt new mandatory identification and limit-setting practices (see Marionneau et al. [25]) by registering as Veikkaus customers.

These recent marketing restrictions have not targeted offshore operators. A significant part of the Finnish gambling market consists of non-licensed offshore operation [26]. In 2023, the Finnish government decided to dismantle the monopoly and open the gambling market to commercial licensed operators by 2027. The primary aim of the proposed law is to reduce harm by channelling consumption and provision of gambling from offshore operators to the regulated market [27]. To achieve the channelling objective, the law proposes significant relaxations on marketing regulations: new forms of marketing will be legalised, including the marketing of online casinos [27]. Consequently, the volume of marketing is likely to increase substantially.

This paper focuses on experiences of gambling marketing in a country with a changing landscape of gambling marketing regulations. We ask what kind of gambling marketing Finns are currently exposed to and what kind of regulatory measures they prefer. To address these questions, we conducted an online survey aimed at individuals who gamble actively.

## Methods

### Data

The data were collected with an online survey that contained questions on background information, recent gambling policy changes in Finland and gambling marketing. Only questions related to background information and gambling marketing were analysed for this study.

The data were collected between August and September 2023. The survey was distributed on social media (Facebook, Twitter, Reddit), popular Finnish online forums and in land-based gambling locations in Southern Finland and Lapland. On social media, we targeted the survey to individuals interested in gambling. Our inclusion criterion was individuals who actively gamble and want to provide their opinions on current issues related to Finnish gambling policy.

In total, 132 individuals completed the survey. Whilst our data collection was based on convenience sampling, we achieved a sample of individuals who gambled regularly and were familiar with the Finnish gambling context.

### Analysis methods

The survey focused on experiences and perceptions of gambling marketing, using multiple-choice and open-ended questions. The survey questions are presented in Table I.

**Table I.** Survey questions.

	Survey question	Multiple-choice	Open-ended
1.	What do you consider to be gambling marketing?	–	⨯
2.	What do you consider to be gambling marketing?	Options presented in Figure 1.	–
3.	Has gambling marketing changed in recent years in your opinion?	Yes No Do not know	–
4.	How has gambling marketing changed in recent years in your opinion?	–	⨯
5.	Have you seen mandatory identification campaigns by Veikkaus recently?	Yes No Do not know	–
6.	What do you think about these mandatory identification campaigns and why?	–	[If yes to question 5]
7.	Have you encountered Veikkaus advertising, marketing or other visibility online?	Yes No Do not know	–
8.	What kind of Veikkaus advertising, marketing or other visibility have you encountered?	–	[If yes to question 7]
9.	Have you encountered offshore provider advertising, marketing or other visibility online?	Yes No Do not know	–
10.	What kind of offshore provider advertising, marketing or other visibility have you encountered?	–	[If yes to question 9]
11.	Many European jurisdictions are tightening regulations on gambling marketing. How should Finland regulate gambling marketing? (multiple choice)	Prohibit marketing Tighten regulations Maintain status quo Liberalise regulations Do not know or other	–
12.	Why?	–	[Depending on the choice in question 11]
13.	How do you think gambling marketing will change in the new licensing system?	–	⨯

We analysed the data by integrating quantitative and qualitative analysis methods. Multiple-choice questions were analysed statistically using bivariate correlations between the multiple-choice questions and socio-economic characteristics, and a chi-square test of independence was performed to examine the relationship between operator types. Quantitative findings are produced with SPSS.

Open-ended questions were analysed qualitatively by using Atlas.ti. While the initial coding categories were based on the survey questions, additional codes were developed when necessary. Eventually, responses were divided into critical (sub-codes including towards marketing, mandatory identification, regulation), neutral (or indifferent to respondents) and positive (harm prevention, consumer protection, free market, etc.).

Results are reported with example quotations. All translations from Finnish to English are by the authors.

### Research ethics

Based on the advice of the Finnish National Board on Research Integrity, this study did not require an ethics evaluation. All participants received information about the study and data protection following the University of Helsinki Data Protection guidelines. All participants gave informed consent to participate in this study.

## Results

### Sample characteristics

Table II presents the sample characteristics. The sample had more male than female participants and it was also skewed towards younger age groups and individuals living in the capital region. Of the participants, 65.2% gambled with the Veikkaus monopoly, 19.7% with offshore providers and 14.4% equally with both. Men were more likely than women to gamble with offshore providers. Based on self-assessment, 9.8% of respondents reported current and 11.4% past gambling problems, with more women reporting current and more men past problems.

**Table II.** Sample characteristics.

Age	*n*	Percentage	*n*	Male	*n*	Female	*n*	Other
18–24	6	4.5%	4	5.3%	2	3.7%		
25–34	32	24.2%	19	25.0%	12	22.2%	1	50.0%
35–49	48	36.4%	27	35.5%	20	37.0%	1	50.0%
50–64	32	24.2%	16	21.1%	16	29.6%		
65–74	13	9.8%	9	11.8%	4	7.4%		
75+	1	0.8%	1	1.3%				
**Total**	132	100%	76	100%	54	100%	2	100%
**Place of residence**								
Capital region	58	43.9%	36	47.4%	22	40.7%		
Other large town	37	28.0%	20	26.3%	16	29.6%	1	50.0%
Small town/countryside	35	26.5%	19	25.0%	15	27.8%	1	50.0%
No information	2	1.5%	1	1.3%	1	1.9%		
**Total**	132	100%	76	100%	54	100%	2	100%
**Gamble predominantly**								
Veikkaus	86	65.2%	41	53.9%	43	79.6%	2	100.0%
Offshore operator	26	19.7%	21	27.6%	5	9.3%		
Both equally	19	14.4%	13	17.1%	6	11.1%		
No information	1	0.8%	1	1.3%				
**Total**	132	100%	76	100%	54	100%	2	100%
**Has gambling been a problem (self-assessment)**								
Yes, currently	13	9.8%	5	6.6%	8	14.8%		
Yes, but not any more	15	11.4%	12	15.8%	3	5.6%		
No	95	72.0%	53	69.7%	41	75.9%	1	50.0%
Does not know/no information	9	6.8%	6	7.9%	2	3.7%	1	50.0%
**Total**	132	100%	76	100%	54	100%	2	100%

Bivariate correlations in Table S1 in the Supplementary material online show statistically significant associations between operator type and gender, age and self-reported gambling problems. Men and younger respondents were more likely to gamble with offshore providers or with both Veikkaus and offshore operators. Those gambling offshore were more likely to report past or current gambling problems.

### Perceptions on forms of marketing

Despite marketing restrictions, our respondents reported widespread exposure to and recognition of different forms of marketing. Open-ended answers showed that most respondents recognised direct advertising, such as television and radio commercials or online and outdoor advertising. Several respondents also expressed a wider perspective on the range of marketing:

‘Ads, logos, news articles, social media influencers’ posts etc. Gambling venues, lottery tickets and sports betting slips at kiosks, electronic gambling machines at supermarkets, information on Veikkaus etc. etc.’ (Female, 35–49, Veikkaus)

‘All marketing aiming to sell gambling. Indirect advertising is difficult to recognise though.’ (Male, 18–24, Veikkaus)

Figure 1 presents results on what our respondents considered as marketing, besides traditional formats of commercial communication. Most perceived social media posts by gambling operators as marketing (81.1%), followed by gambling-related social media posts by influencers (75.8%). Sports sponsorship was recognised as marketing by 68.1% of the respondents. Conversely, less than 30% of respondents considered the pricing of gambling products as marketing. Less than 50% viewed product design and product information as marketing. Of the respondents, 2.3% did not consider any of the options as marketing; 20.4% ticked all options.

**Figure 1.**
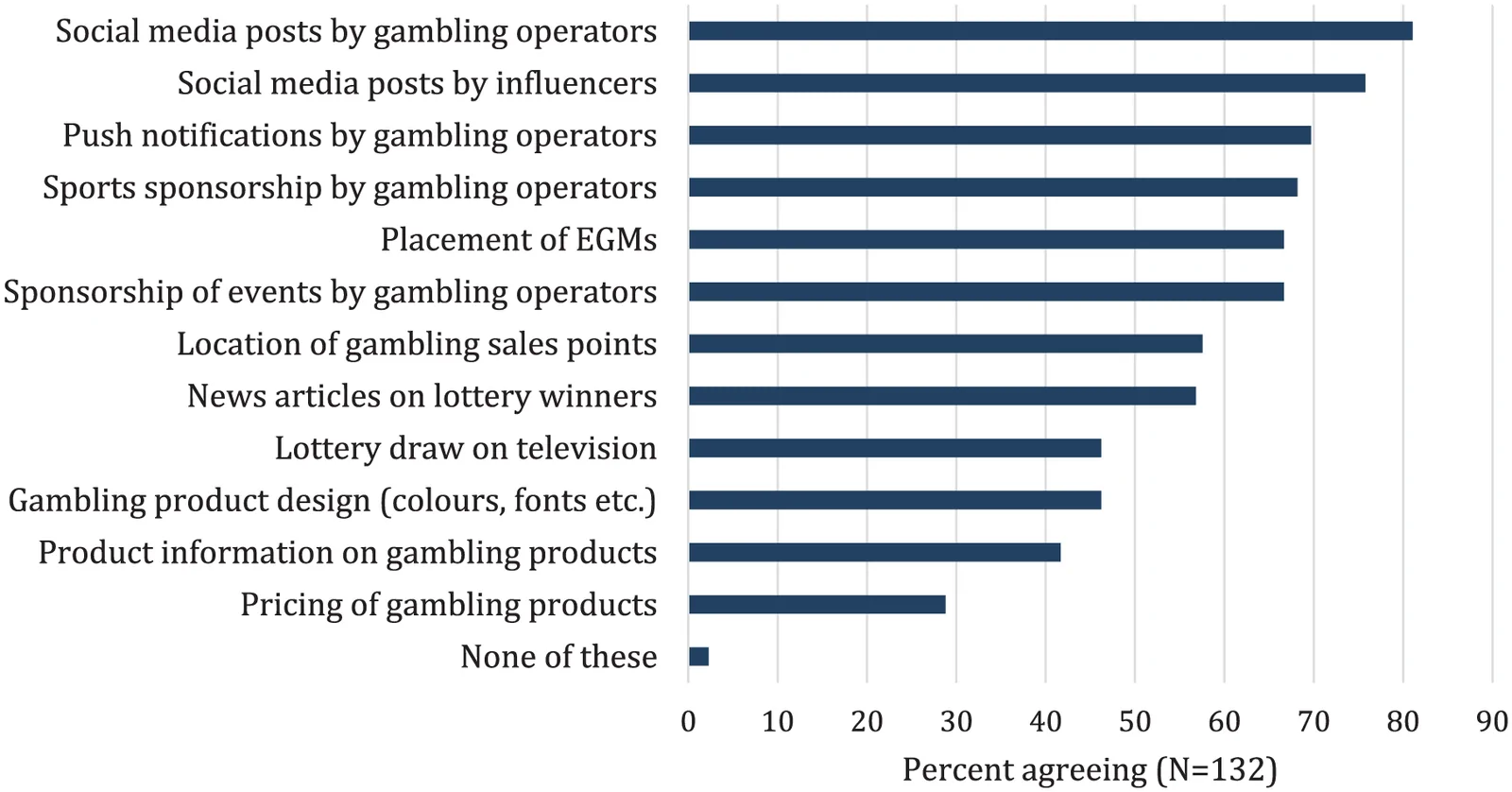
What is considered as marketing by Finnish individuals who gamble actively. EGM = Electronic Gambling Machine.

### Experiences of advertising and marketing

Since the current Finnish gambling marketing restrictions apply to only the regulated market, we analysed exposure based on whether respondents gambled exclusively with Veikkaus or with unlicensed operators (either exclusively or in addition to Veikkaus). Table III shows that, overall, 70.1% of participants had seen online marketing by Veikkaus and 79.6% by offshore providers. Table IV shows statistically significant associations for exposure to offshore operator advertising, exposure to Veikkaus campaigning and observed changes in marketing. We found no significant differences in exposure to Veikkaus advertising depending on type of operator.

**Table III.** Cross-tabulation and chi-square of experiences of marketing between operator types.

	All		Veikkaus		Offshore and both equally		*p*
	*n*	%	*n*	%	*n*	%	
**Seen Veikkaus marketing online (*n* = 97)**							0.570
Yes	68	70.1	44	72.1	24	66.7	
No	29	29.9	17	27.9	12	29.9	
**Seen marketing online by other operators (*n* = 108)**							0.006
Yes	86	79.6	47	71.2	39	92.9	
No	22	20.4	19	28.8	3	7.1	
**Seen Veikkaus campaigns for mandatory identification (*n* = 118)**							0.030
Yes	101	85.6	74	90.2	27	75.0	
No	17	14.4	8	9.8	9	25.0	
**Has gambling marketing changed in the past years (*n* = 89)**							0.015
Yes	64	71.9	48	80.0	16	55.2	
No	25	28.1	12	20.0	13	44.8	

**Table IV.** Cross-tabulation and chi-square of perceptions on gambling marketing regulations between operator types.

	**All**		**Veikkaus**		**Offshore or both**		***p***
	*n*	%	*n*	%	*n*	%	
**Perceptions on gambling marketing regulations (*n* = 109)**							0.064
Liberalise regulations	17	15.6	9	12.5	8	21.6	
Maintain status quo	26	23.9	22	30.6	4	10.8	
Tighten regulations	30	27.5	21	29.2	9	24.3	
Prohibit marketing	36	33.0	20	27.8	16	43.2	

Table S1 in the Supplementary material presents statistically significant correlations between exposure to offshore marketing and both age and gender, with men and younger respondents reporting higher exposure. Exposure to offshore advertising was also positively correlated with exposure to Veikkaus’ marketing and with perceived changes in the marketing landscape.

As Veikkaus had recently reduced its marketing budget, we asked whether participants had noticed any changes in marketing. Approximately half of the respondents could identify changes in Veikkaus marketing. However, and despite stricter restrictions, most respondents noted that marketing had increased or become more aggressive:

‘There is a constant influx of advertising on social media, email, text messages and even TV channels.’ (Male, 25–34, Veikkaus/offshore)

Many noted that this increase was primarily due to increased visibility of offshore marketing on the internet and social media, while some also reported more aggressive advertising by Veikkaus.

Offshore marketing, overall, was described as aggressive and unethical. Experiences with offshore advertising included online casino advertisements on social media, streaming platforms and banners. Some mentioned frequently receiving offshore advertising via email or text messages. Marketing of offshore gambling was overall considered harmful:

‘Online casino advertising by foreign operators seems a bit questionable sometimes. Big promises, but they don’t seem to be too interested in preventing risky gambling.’ (Male, 50–64, Veikkaus)

Veikkaus’ marketing was described as less aggressive, but highly prevalent in Finnish society. Many recalled advertisements on the lottery and jackpots, news articles about big wins, social media posts and, most commonly, campaigns urging customers to identify when gambling. Of the respondents, 85.6% had seen these identification campaigns, with only 14.4% not recalling them. Among those gambling with Veikkaus exclusively, 90.2% recalled the campaign.

Opinions about the mandatory identification campaigns were split. Around two-thirds of all respondents considered the campaign to be informative, positive, or had a neutral stance. Many appreciated the identification system and felt that advertising was justified:

‘I think in this case information is a good thing, even though it is still advertising gambling.’ (Male, 18–24, self-reported gambling problem, Veikkaus/offshore)

‘It’s good that they provide information about it not being possible to gamble without identification.’ (Female, 50–64, Veikkaus)

A third of the respondents were critical of the campaign. They expressed annoyance at the frequency of advertising and the celebrities involved in the campaign. For some, the campaign was a waste of money or only a form of whitewashing the company brand:

‘Veikkaus is trying to be so responsible without really doing anything.’ (Male, 25–34, Veikkaus)

### Views on gambling regulation

Owing to the recent marketing restrictions and the forthcoming licensing system, our respondents were well-placed to evaluate what they consider to be the most effective ways to regulate marketing. Of our respondents, 60.5% wanted to either prohibit gambling marketing (33.0%) or tighten existing regulations (27.5%). Those gambling offshore (43.2%) were more supportive of prohibiting gambling marketing than those gambling with Veikkaus only (27.8%). Only 15.6% of respondents supported a liberalisation of regulations. There was no statistically significant difference between operator types.

In the open-ended comments, participants provided further views on how they thought gambling marketing should be best regulated. Amongst those who wanted to prohibit or restrict gambling marketing, comments highlighted a need to align gambling marketing regulations with those on tobacco and nicotine products. Many noted that restrictions would protect vulnerable populations and those with problem gambling:

‘It’s irresponsible. Addiction is a serious problem. Young people are especially susceptible to content marketing on social media.’ (Female, 25–34, self-reported gambling problem, Veikkaus)

‘Gambling should not be visibly available or marketed. Just like tobacco.’ (Male, 18–24, self-reported gambling problem, offshore)

Those who wanted to maintain the status quo suggested that advertising ‘makes no difference on how much people with gambling problems gamble’ (male, 35–49, Veikkaus) or emphasised that advertising did not bother them personally. Some highlighted that while they wanted marketing regulations to remain the same, they should ‘also apply to the new licensed operators’ (male, 35–49, Veikkaus).

Amongst those who wanted to liberalise marketing regulations, some emphasised individual responsibility and freedoms:

‘We are a free nation in a free country with the right to choose for ourselves. ’(Female, 50–64, Veikkaus)

Others highlighted benefits to consumers in terms of bonus offers, or benefits of marketing revenue to sports and other events:

‘Hopefully the gambling industry gets to sponsor more sports and culture.’ (Male, 25–34, offshore)

Finally, we asked the participants to evaluate how gambling marketing will change in the new licensing system. While some were delighted with an additional source of income for sports, most feared a significant increase in the volume of marketing. Many used expressions such as ‘an explosion’ or ‘the wild west’.

‘I am quite concerned. I think that marketing will increase and become increasingly versatile.’ (Male, 25–34, Veikkaus)

## Discussion

This study has investigated perceptions and experiences of gambling marketing among Finnish individuals during a time of a changing gambling marketing landscape. Gambling marketing regulations were tightened in Finland in 2021. However, despite this, our results showed that marketing by both the monopoly and offshore providers was considered extensive: participants were widely exposed to different forms of marketing regardless of the operator. Furthermore, most felt that marketing was becoming more aggressive. This is not surprising as marketing is vital for customer acquisition and retention especially in the global online market where even monopolies must compete with offshore provision.

Restrictions on Veikkaus marketing might have reduced traditional forms of commercial communication. We found that while around 70% of respondents reported seeing Veikkaus marketing online, 85.6% recalled the company’s campaigns for mandatory identification. Although the campaign was presented as a public health initiative encouraging consumers to identify and thus better prevent harm, our respondents regarded it as marketing. Previous research has similarly argued that this type of ‘responsibility messaging’ by commercial companies frequently includes brand names and logos, further enhancing brand recognition [15,28] or even intentions to gamble [14]. Responsibility messaging can therefore be leveraged by gambling companies as a form of marketing, alongside other less recognisable marketing, such as social media content or promotion of charity work [13,16,29].

Of the respondents, 60% wanted to restrict gambling marketing regulations to a greater extent than at present. This finding is somewhat inconsistent with results from the Finnish population study of 2023, in which 73% considered Veikkaus marketing satisfactory [3]. In our study, only 23.9% of respondents were content with the status quo. The difference might be due to different survey questions and sampling methods. Instead of focusing solely on Veikkaus’ marketing, we also inquired about offshore gambling marketing. As a rule, respondents in our study considered offshore marketing more negatively. Additionally, our sample consisted of an active gambling population that is also likely to be highly exposed to extensive gambling marketing. High exposure may lead to more critical opinions. Existing research also shows a connection between exposure to gambling marketing and increased gambling consumption and harms [5,6,10,11].

Several European jurisdictions are currently restricting gambling marketing to address the emerging public health threat it poses. Countries such as Italy, Belgium, Latvia, Lithuania and Spain have already prohibited most or all forms of gambling marketing. In Finland, Veikkaus also reduced its marketing in the early 2020s, while wider visibility was gained through campaigns for mandatory identification. Our results therefore have concrete parallels with efforts in other European jurisdictions to regulate and reduce the negative effects of gambling marketing. Despite reductions in its marketing, Veikkaus’ visibility has remained high. This suggests, similarly to results obtained in Belgium [30], that partial marketing restrictions may lead operators to leverage remaining channels to maintain visibility. Thus, total prohibitions are likely to be a more effective measure. A prohibition on gambling marketing also found support amongst our respondents.

The new licensing system is set to come into effect in Finland by 2027. The system change will have a further impact on the Finnish gambling marketing landscape. As also feared by many of our participants, the volume of marketing is likely to accelerate as new licence holders compete for market shares. The negative impacts of gambling marketing on public health are also likely to increase. Of our respondents, 60.5% preferred tight restrictions or a full ban on gambling marketing. Prioritising public health over corporate interests in marketing regulations is crucial to preventing severe gambling-related harm to society.

### Limitations

Our data are based on convenience sampling. Therefore, the results should be considered exploratory rather than representative of the Finnish population. However, convenience sampling has allowed us to target the survey to individuals who gamble actively. This group is often neglected in gambling studies that focus on population samples or help-seekers. Our dataset was also relatively small (*N*=132), limiting possibilities for statistical analyses. An online survey is not optimal for in-depth qualitative analysis: most open-ended questions received relatively short responses (1–2 sentences) and there was no possibility for follow-up questions. However, combining qualitative and quantitative data has produced a more nuanced picture of experiences with gambling marketing in a context with recent regulatory restrictions.

### Recommendations for further studies

Further studies should focus on the impact of different forms of marketing on harms and consumption. Gambling marketing consists of a wide range of practices that are likely to have diverse impacts across population groups. It would also be important to conduct longitudinal research on how marketing evolves in Finland as the system changes from a monopoly to a licensing model. This would allow tracking and understanding how different policy choices can impact individual experiences and marketing exposure.

## Conclusion

This study has shown that despite recent regulatory restrictions on gambling marketing in Finland, individuals who gamble actively are exposed to widespread and increasingly aggressive gambling marketing by the monopoly provider and by offshore operators. Gambling marketing ranges from direct and indirect promotional communications to sponsorship, social media content and collaborations, and even leveraging public health messaging for company visibility. In Finland, the volume of gambling marketing is expected to increase significantly following the new licensing system and a relaxed regulatory framework. Yet, our results show that few individuals want more gambling marketing. Thus, regulations must be comprehensive to reduce negative impacts on public health. Otherwise, industry actors are likely to use existing loopholes to maintain visibility.

## Supplemental Material

sj-docx-1-sjp-10.1177_14034948251394913 – Supplemental material for Gambling marketing in Finland: Experiences of exposure and regulation among Finnish individuals who gamble regularly Supplemental material, sj-docx-1-sjp-10.1177_14034948251394913 for Gambling marketing in Finland: Experiences of exposure and regulation among Finnish individuals who gamble regularly by Paula P. Jääskeläinen, Eveliina V. Väkeväinen and Virve K. Marionneau in Scandinavian Journal of Public Health
