# Supplementary material for: Gambling marketing in Finland: Experiences of exposure and regulation among Finnish individuals who gamble regularly
Source: Scand J Public Health. 2025 Dec 17;54(8):817–25. doi: 10.1177/14034948251394913 (PMC13710722; doi:10.1177/14034948251394913)
Supplement: Supplementary file 1 [file sj-docx-1-sjp-10.1177_14034948251394913.docx]

SUPPLEMENTARY FILE

**Table S1. Bivariate correlations for study variables**

| **Variable** | **N** | **1.** | **2.** | **3.** | **4.** | **5.** | **6.** | **7.** | **8.** | **9.** | **10.** |
| --- | --- | --- | --- | --- | --- | --- | --- | --- | --- | --- | --- |
| 1. Gender ^a^ | 130 | - |  |  |  |  |  |  |  |  |  |
| 2. Age | 132 | -,01 |  |  |  |  |  |  |  |  |  |
| 3. Place of residence ^b^ | 130 | ,06 | -,12 |  |  |  |  |  |  |  |  |
| 4. Gamble predominantly ^c^ | 131 | **,26^**^** | **-,25^**^** | ,03 |  |  |  |  |  |  |  |
| 5. Problem, self-assessment ^d^ | 123 | ,04 | **-,20^*^** | ,05 | **,48^**^** |  |  |  |  |  |  |
| 6. Perceptions of regulation ^e^ | 109 | -,03 | -,17 | **,20^*^** | ,08 | **,24^*^** |  |  |  |  |  |
| 7. Seen Veikkaus’ marketing ^d^ | 97 | ,08 | -,18 | ,18 | -,06 | -,13 | -,07 |  |  |  |  |
| 8. Seen other operators' marketing ^d^ | 108 | **,21^*^** | **-,24^*^** | ,08 | **,26^**^** | ,18 | **,20^*^** | **,23^*^** |  |  |  |
| 9. Seen mandatory identification ^d^ | 118 | -,06 | ,03 | ,06 | **-,20^*^** | -,03 | -,16 | **,32^**^** | ,15 |  |  |
| 10. Marketing change ^d^ | 89 | -,03 | ,03 | ,11 | **-,26^*^** | -,03 | **,24^*^** | ,10 | **,27^*^** | -,04 | - |

^a^ 1 = female, 2 = male

^b^ 1 = small town or countryside, 2 = other large town, 3 = capital region

^c^ 1 = Veikkaus and 2 = offshore operator or both equally

^d^ 1 = no and 2 = yes

^e^ 1 = liberalize, 2 = maintain, 3 = tighten, 4 = prohibit

* *p* < .05. ** *p* < .01.
